# Investigation on the IL-18 -607A/C and -137C/G on the susceptibility of ischemic stroke

**DOI:** 10.12669/pjms.311.5997

**Published:** 2015

**Authors:** Jin-he Shi, Li-dan Niu, Xi-yan Chen, Jing-yu Hou, Ping Yang, Guang-peng Li

**Affiliations:** 1 Jin-he Shi, Department of Emergency, The First Affiliated Hospital of Xinxiang Medical University, Weihui, China.; 2 Li-dan Niu, Department of Emergency, The First Affiliated Hospital of Xinxiang Medical University, Weihui, China.; 3 Xi-yan Chen, Department of Emergency, The First Affiliated Hospital of Xinxiang Medical University, Weihui, China.; 4 Jing-yu Hou, Department of Emergency, The First Affiliated Hospital of Xinxiang Medical University, Weihui, China.; 5 Ping Yang, Department of Emergency, The First Affiliated Hospital of Xinxiang Medical University, Weihui, China.; 6 Guang-Peng Li, Department of Emergency, The First Affiliated Hospital of Xinxiang Medical University, Weihui, China.

**Keywords:** IL-18, Ischemic stroke, Polymorphism

## Abstract

**Objective:**

We conducted a case-control study with 322 cases and 322 controls to assess the role of the two common SNPs in the promoter of IL-18 gene.

**Methods:**

Polymerase chain reaction restriction fragment length of polymorphism (PCR-RFLP) was taken to genotype -607A/C and -137C/G in the promoter of the IL-18 gene.

**Results:**

By comparing cases and control subjects, we found that IS cases were more likely to have higher BMI, higher proportion of hypertension, and have higher proportion of smokers and drinkers. We found that IL-18 -607CC genotype (OR=1.70, 95% CI=1.03-2.81) and C allele (OR=1.26, 95% CI=1.01-1.58) were significantly more frequent in IS patients when compared with AA genotype. We did not find significant association between IL-18 -607A/C gene polymorphism and BMI, hypertension, smoking and drinking on the risk of IS.

**Conclusion:**

Our study suggests that polymorphisms in IL-18 -607A/C can influence the development of IS, and this gene polymorphism is associated with risk of IS in a Chinese population.

## INTRODUCTION

Stroke is a leading cause of death, and this disease causes long-term disability in the world. Stroke causes a great burden for patients and their families because of lack of illustrated etiology and effective treatments.^1^ It is estimated that more than 80% of the stroke cases are ischemic stroke (IS) in origin. It is well known that IS is a mutifactorial disease with many environmental and genetic factors. Environmental factors such as hypertension, hyperlipidemia, smoking and drinking habits can increase the risk of IS, and many genetic factors also play an important role in the development of IS.^2^ Increasing evidences showed that inflammatory response have a role in the susceptibility to IS.^3^ Since inflammatory factors are one of the candidate genes for the risk of IS, the role of many inflammatory gene variations has been widely investigated in recent several years.

Several previous studies have shown that several certain polymorphisms in inflammatory genes have an important role in the development of IS, such as intereleukin-6 (IL-6), transforming growth factor (TGF-B1), interleukin-1 receptor antagonist(IL-1Ra), IL-1a and IL-1B and tumor necrosis factors as well as lymphotoxin-a.^4^^-^^8^ IL-18 is a member of the IL-1 superfamily, and this gene is a pleiotropic pro-inflammatory cytokine and has a role in the inflammatory cascade.^9^ Two previous studies have reported that higher level of IL-18 is associated with increased risk of IS.^10^^,^^11^

It is reported that the IL-18 gene is located on the region of chromosome 11q22.2-q23.3.^12^^,^^13^ There were two common gene polymorphisms, -607A/C(rs1946518) and -137C/G(rs187238), in the promoter of IL-18 gene. Previous studies have shown that C allele of IL-18-607A/C and G allele of IL-18-137C/G could affect the IL-18 higher transcription and its protein production.^14^^,^^15^ Previous studies have also shown that IL-18 gene polymorphisms are correlated with risk of cardiovascular disease.^16^^,^^17^ However, few studies have reported the association between IL-18 polymorphisms and risk of IS.

In order to assess the role of the two common SNPs of the promoter of IL-18 gene in the risk of IS, we conducted a case-control study with 322 cases and 322 controls in a Chinese population.

## METHODS

***Subjects:*** Our study included 322 patients who were first onset of IS, and all the patients with IS were selected from the First Affiliated Hospital of Xinxiang Medical University between January 2011 and January 2013. The diagnosis of IS was based on the clinical, cardiac, ultrasound and brain Magnetic Resonance Imaging (MRI) or Computed Tomography (CT) tests. The diagnosis criteria was according to World Health Organization, and that is rapidly development of clinical signs of focal or global disturbance of cerebral function lasting>24 hours with no apparent cause but vascular origin. Cases who had type 2 diabetes, transient ischemic attacks, intracranial hemorrhage, congestive heart failure, postseizure palsy and brain tumor as well as asthma were excluded from this study.

The controls were frequency-matched with cases by age and sex. 322 control subjects were collected from the heath check-up center of the First Affiliated Hospital of Xinxiang Medical University, who came to our hospital for regular health check-up. An informed consent was obtained from all participants before enrolling into our study. The protocol of this study was approved by the First Affiliated Hospital of Xinxiang Medical University.

The age, sex, Body Mass Index (BMI), smoking and drinking habits, hypertension and diabetes were collected by a self-designed questionnaire. The BMI was calculated by weight divided by height squared. Hypertension was assessed according to hypertension guidelines from World Health Organization.

***Genotyping assays:*** A 5mL sample of venous blood was collected from each IS case and control subject after enrolling into our study. The blood samples were kept at -20C until use, and EDTA 0.5 mg/ml was used to be anticoagulant for blood samples. Genomic DNA was extracted from the whole blood using a TIANamp blood DNA kit (Tiangen Biotech, Beijing, China) according to manufacturer’ instructions.

Polymerase chain reaction restriction fragment length of polymorphism (PCR-RFLP) was taken to genotype -607A/C and -137C/G in the promoter of the IL-18 gene. The PCR primers of IL-18 -607A/C & -137C/G were designed using primer 5.0 software according to the manufacturer’ instructions. Primers for -607A/C were 5’-GTTGCAGAAAGTGTAAAAATTATTAC-3’ (Forwards) and 5’-GTTGCAGAAAGTGTAAAAATTATTAA-3’ (Reverse), and primers for -137C/G were 5’-CCCCAACTTTTACGGAAGAAAAG-3’ and 5'- CCCCAACTTTTACGGAAGAAAAC-3’.

Amplification was conducted using the following processes: beginning with an initial denaturation for 5 minutes at 95°C, followed by 35 step cycles of denaturation at 95°C for 30s, annealing at 62°C for 30s, and extension at 72°C for 30s, and a final extension was taken at 72°C for 10 minutes. PCR products of IL-18 gene were assessed by 1.0% agarose gel electrophoresis and ultraviolet light. In order to perform quality control, we randomly selected 5% of IS cases and controls to repeat genotype, and we found the reproducibility was 100%.

***Statistical Analysis:*** Continuous variables were shown as mean ± SD, and categorical variables were shown as frequencies and percentage (%). The continuous and categorical variables between cases and controls were compared by the χ^2^-test or student *t* test. A χ^2^-test was taken to assess the Hardy–Weinberg equilibrium of genotype distributions of control group. After adjusting potential risk factors of IS, a multivariate logistic regression analysis was performed to compare the genotype distributions of -607A/C and -137C/G in the promoter of the IL-18 gene between case and control groups. The results were expressed by an odds ratio (OR) and 95% confidence interval (95% CI). The homozygous genotypes of IL-18 -607A/C and -137C/G genotypes were used to be reference group. Two-sided* P*-values were taken to use in our study and a *P*-value<0.05 was defined as statistically significant. All statistical analyses were performed using STATA 9.0 software.

## RESULTS

The demographic and lifestyle characteristics of IS patients and control subjects were shown in Table-I. There was no significant difference in terms of sex and age between cases and controls. By comparing characteristics between IS cases and control subjects, we found that IS cases were more likely to have higher BMI, higher frequency of hypertension, and have higher frequency of smokers and drinkers than control subjects.

**Table-I:** Distributions of the demographic & lifestyle characteristics

**Characteristics**	**Cases**	**%**	**Controls**	**%**	***t*** ** or χ** ^2^	**p value**
Age, years						
Mean +SD, years	62.4±9.3		61.8±10.6			
<60	149	46.27	170	52.80		
≥60	173	53.73	152	47.20	2.74	0.10
Sex						
Male	204	63.35	204	63.35		
Female	118	36.65	118	36.65	0.00	1.00
BMI(kg/m^2^)						
<25	207	64.29	233	72.36		
≥25	115	35.71	89	27.64	4.85	0.03
Diabetes						
No	73	22.67	59	18.32		
Yes	249	77.33	263	81.68	1.87	0.17
Hypertension						
No	181	56.21	82	25.47		
Yes	141	43.79	240	74.53	62.99	<0.001
Smoking						
Non-smoker	92	28.57	56	17.39		
Current or ex-smoker	230	71.43	266	82.61	1.9	<0.001
Drinking						
No	105	32.61	133	41.30		
Yes	217	67.39	189	58.70	5.23	0.02

The genotype and allele frequencies of IL-18 -607A/C and -137C/G are shown in Table-II. The genotype and allele frequencies of the IL-18 -607A/C and -137C/G in controls did not deviate from Hardy-Weinberg equilibrium in the case and control groups. There was significant difference in the allele frequencies in terms of -607A/C between cases and controls (*P*<0.05) (Table-II).

IL-18 -607CC genotype (OR=1.70, 95% CI=1.03-2.81) and C allele (OR=1.26, 95% CI=1.01-1.58) were significantly more frequent in IS patients when compared with AA genotype (Table-II). However, we did not find significant association of IL-18 -137C/G gene polymorphism with risk of IS.

**Table-II:** Associated of IL-18 genotypes with risk of IS

**IL-18 polymorphism**	**Cases**	**%**	**Controls**	**%**	**p value**	**OR(95%CI)** ^1^
-607A/C genotype						
AA	54	16.77	71	22.05	-	1.0(Ref.)
AC	180	55.9	183	56.83	0.22	1.29(0.84-1.99)
CC	88	27.33	68	21.12	0.03	1.70(1.03-2.81)
Allele						
A	288	44.72	325	50.47	-	1.0(Ref.)
C	356	55.28	319	49.53	0.04	1.26(1.01-1.58)
-137C/G genotype						
CC	11	3.42	18	5.59	-	1.0(Ref.)
CG	81	25.16	84	26.09	0.27	1.58(0.66-3.94)
GG	230	71.43	220	68.32	0.17	1.71(0.75-4.10)
Allele						
C	103	15.99	120	18.63	-	1.0(Ref.)
G	541	84.01	524	81.37	0.21	1.20(0.89-1.62)

1. Adjusted for sex, age, BMI, diabetes, hypertension, smoking and drinking

We further investigated the association between IL-18 -607A/C gene polymorphism and BMI, hypertension, smoking and drinking on the risk of IS (Table-III). Stratified analysis showed that an increased risk of IS was associated with those carrying IL-18 -607AC+CC, even in those with higher BMI, hypertension, smoking and drinking habits. However, no statistical significant difference was found between them, and no significant interaction between IL-18 -607A/C gene polymorphism and BMI, hypertension, smoking and drinking on the risk of IS.

**Table-III:** Association between IL-18 -607A/C gene polymorphism and demographic characteristics on the risk of IS.

**Characteristics**	**-607AA**		**-607AC+CC**		**OR(95%CI)** ^1^	**p value**
	Cases	Control	Cases	Control		
BMI(kg/m^2^)						
<25	36	51	171	182	1.33(0.81-2.21)	0.24
≥25	18	20	97	69	1.56(0.72-3.38)	0.21
Hypertension						
No	31	22	150	60	1.77(0.90-3.45)	0.07
Yes	23	49	118	191	1.32(0.74-2.39)	0.32
Smoking						
Non-smoker	13	11	79	45	1.49(0.55-3.92)	0.38
Current or ex-smoker	41	60	189	206	1.34(0.84-2.15)	0.19
Drinking						
No	19	28	86	105	1.21(0.60-2.45)	0.56
Yes	35	43	182	146	1.53(0.90-2.60)	0.09

1. Adjusted for sex and age

## DISCUSSION

The etiologies of IS are highly complicated and not well understood, and both environmental and genetic factors play a role in the development of IS, such as smoking, drinking, hypertension, diabetes and some genetic polymorphisms. Previous studies indicated that several certain polymorphisms in inflammatory genes can influence the risk of IS, such as intereleukin-6 (IL-6), transforming growth factor (TGF-B1), interleukin-1 receptor antagonist (IL-1Ra), IL-1a and IL-1B and tumor necrosis factors.^4^^-^^8^ The current study showed that IL-18 -607A/C polymorphisms can influence the risk of IS.

Genetic polymorphisms of cytokines can influence the expression or function of these cytokines, and polymorphisms in the genes can regulate the inflammatory mechanisms that play an important role in the pathology and development of IS. Previous study has shown that genetic polymorphisms of IL-18 -607A/C and -137C/G in the promoter IL-18 gene may affect the immune response, and can have a role in the risk of several kinds of diseases, such as tuberculosis, cancers, coronary artery disease and stroke.^16^^,^^18^^-^^21^

IL-18 -607A/C and -137C/G are located at the promoter of IL-18 gene, and previous studies have showed that the -607A allele and -137C allele are correlated with higher expression of IL-18.^22^^,^^23^ IL-18 has a role in causing spread and instability of atherosclerotic plaque, and previous studies have shown that IL-18 gene variations are associated with vascular changes in the carotid artery and associated with risk of coronary artery disease.^16^^,^^24^^,^^25^ Altering expression of IL-18 can cause the proliferation of human aortic smooth muscle cell, and thus induce the progress of atherosclerosis.^26^^,^^27^ Moreover, IL-18 is also involved in neuroinflammation in hypoxic-ischemic brain injuries.^28^ Only two previous clinical studies have shown that IL-18 gene polymorphisms are associated with development of IS.^20^^,^^21^ Zhang et al. investigated the association between IL-18 -607A/C and -137C/G and risk of ischemic stroke, and reported that -607C allele was associated with an increased risk of IS, and –137G allele was associated with increased risk of larger artery atheroscherosis.^20^ Lu et al. reported that IL-18-607C/A promoter polymorphism can influence the risk of IS, and C allele was correlated with an increased risk.^21^ The results of our study were in line with previous studies, our study reported that 607C allele was associated with increased risk of IS.

Several strengths can be considered in our study. First, the diagnosis of IS was used a rigorous method. The diagnosis of IS was based on the clinical, cardiac, ultrasound and brain Magnetic Resonance Imaging (MRI) or Computed Tomography (CT) tests. Second, a relative larger sample size was taken in our study, which enhances the statistical power to find the difference between groups. Some limitations also should be considered in our study. First, our study is a case-control design, and thus a selection bias could not be avoided for the case and control groups. Second, this study might be applied only to our populations due to ethnicity differences. Third, other inflammatory gene polymorphisms may have a role in the development of IS, and may have interaction with IL-18 gene polymorphisms. Therefore, further large sample studies with more ethnicities are needed.

In conclusion, our study suggests that polymorphisms in IL-18 -607A/C can influence the development of IS, and this gene polymorphism is associated with risk of IS in a Chinese population. Our study supports the hypothesis that IL-18 gene polymorphism is associated with risk of IS, which suggests that further therapeutic studies should be conducted to reduce the activity of IL-18 to prevent the risk of IS.

## Author’s Contributions:

**JHS:** Conceived, designed and did statistical analysis & editing of manuscript.

**LDN, XYC, JYH, PY & GPL:** Did data collection and manuscript writing.
